# Vacuolin-1 enhances RA-induced differentiation of human myeloblastic leukemia cells: evidence for involvement of a CD11b/FAK/LYN/SLP-76 axis subject to endosomal regulation that drives late differentiation steps

**DOI:** 10.1186/s13578-022-00911-6

**Published:** 2022-11-03

**Authors:** Kaiyuan Zhu, Noor Kazim, Jianbo Yue, Andrew Yen

**Affiliations:** 1 Department of Biomedical Sciences, Cornell University, Ithaca, NY USA; 2 Department of Biomedical Sciences, City University of Hong Kong, Hong Kong, China; 3 Division of Natural and Applied Sciences, Synear Molecular Biology Lab, Duke Kunshan University, Kunshan, China; 4 City University of Hong Kong Shenzhen Research Institute, ShenZhen, China

**Keywords:** Vacuolin-1, Retinoic acid, HL-60 cells, CD11b/FAK/LYN/SLP-76 axis, NUMB, Differentiation therapy, Leukemia.

## Abstract

**Background:**

Retinoic acid(RA), an embryonic morphogen, regulates cell differentiation. Endocytosis regulates receptor signaling that governs such RA-directed cellular processes. Vacuolin-1 is a small molecule that disrupts endocytosis, motivating interest in its effect on RA-induced differentiation/arrest. In HL-60 myeloblastic-leukemia cells, RA causes differentiation evidenced by a progression of cell-surface and functional markers, CD38, CD11b, and finally reactive oxygen species(ROS) production and G1/0 cell cycle arrest in mature cells.

**Results:**

We found that Vacuolin-1 enhanced RA-induced CD11b, ROS and G1/0 arrest, albeit not CD38. Enhanced CD11b expression was associated with enhanced activation of Focal Adhesion Kinase(FAK). Adding vacuolin-1 enhanced RA-induced tyrosine phosphorylation of FAK, Src Family Kinases(SFKs), and the adaptor protein, SLP-76, expression of which is known to drive RA-induced differentiation. Depleting CD11b cripples late stages of progressive myeloid differentiation, namely G1/0 arrest and inducible ROS production, but not expression of CD38. Loss of NUMB, a protein that supports early endosome maturation, affected RA-induced ROS and G1/0 arrest, but not CD38 expression.

**Conclusion:**

Hence there appears to be a novel CD11b/FAK/LYN/SLP-76 axis subject to endosome regulation which contributes to later stages of RA-induced differentiation. The effects of vacuolin-1 thus suggest a model where RA-induced differentiation consists of progressive stages driven by expression of sequentially-induced receptors.

**Supplementary Information:**

The online version contains supplementary material available at 10.1186/s13578-022-00911-6.

## Introduction

Acute myeloid leukemia (AML) is a type of blood cancer caused by the inhibition of differentiation of bone marrow hematopoietic stem cells. It manifests as arrest of the normal development of blood cells of the myeloid series. It is largely incurable by current therapeutic modalities that use cytotoxic chemotherapy. In the United States, about 20,000 people are diagnosed with AML every year, with 5 patients per 100,000 people, and about 80% of patients die from AML [1]. One of the leading reasons for its high mortality rate is that AML often occurs in elderly people. The median age of AML patients is 70 years old. The standard course of clinical treatment for AML patients involves aggressive cytotoxic chemotherapy combined with allogeneic bone marrow transplantation. However, for the elderly this is not well tolerated [2, 3]. Therefore, it is of great clinical significance to develop new therapies with less host toxicity for AML.

Inducing leukemia cell differentiation is an important alternative therapy for the treatment of AML. This differentiation therapy is not as toxic as traditional chemotherapy with cytotoxic drugs and may be better tolerated by elderly patients. Retinoic acid (RA) is an active metabolite of vitamin A. It and its metabolites bind the retinoic acid receptor (RAR) and retinoid X receptor (RXR), which are ligand-activated transcription factors that regulate gene transcription to cause cell differentiation [4]. RA is used clinically to treat acute promyelocytic leukemia (APL), which is a subtype of AML. APL accounts for about 10 -15% of AML patients. Unlike other types of AML with high mortality, about 90% of APL patients can be put in remission after treatment with RA and arsenic trioxide-based differentiation therapy [5]. APL is characterized by accumulation of promyelocytic cells due to the t(15; 17) chromosomal translocation. The t(15; 17) chromosomal translocation results in a promyelocytic leukemia (PML)-RA receptor alpha (RARα) fusion protein, which prevents the transcription of pro-differentiation genes. Therapeutic pharmacological doses of RA can bind PML/RARα to activate transcription of downstream pro-differentiation genes with degradation of the fusion protein when used with arsenic trioxide [6]. However, RA-induced cell differentiation therapy is ineffective for non-APL AML. Combination therapies with RA and other agents rationally determined from mechanistic insights would ergo potentially make differentiation therapy a viable treatment for non-APL AML.

The HL-60 cell is a classic cell line used to study the differentiation of AML cells. It comes from a 36-year-old patient with AML. It does not have the t(15; 17) chromosomal translocation, so it is not an APL cell [7]. However, like APL cells, HL-60 cells can be induced to differentiate into mature granulocytes by RA in a process marked by progressive expression of cell surface markers, CD38, then CD11b, and finally a functional marker, inducible oxidative metabolism (production of ROS), and G1/0 cell cycle arrest characterizing mature cells [8]. Therefore, studying the signaling pathways that RA uses to cause HL-60 differentiation and screening for drugs that promote HL-60 differentiation will effectively inform the development of differentiation therapy for AML patients. RA-induced HL-60 cell differentiation and growth arrest is driven by a signaling machine, signalosome [9–18] putatively regulated by membrane receptors, including CD38, BLR1, and c-FMS. It is activated by RA-induced FGR expression, and it includes signaling molecules historically associated with promoting MAPK pathway signaling such as LYN and SLP-76. The RAF/MEK/ERK axis is imbeeded in the signalosome. The signaling is prolonged in contrast to the transient character of mitogenic signaling and causes nuclear translocation/enrichment of RAF and other components to drive the progression of differentiation.

Integrin-protein tyrosine kinase 2 (FAK, aka Focal Adhesion Kinase) is one of the well-known activators of Src kinases. The β-integrin on the cell membrane recruits FAK resulting in the autophosphorylation of the FAK Y397 site to activate it [19, 20]. Y397 FAK phosphorylation then provides a high-affinity binding site for the Src kinase family members. Src binds to FAK and undergoes Y416 site phosphorylation and conformational changes to become activated [21]. The connections between LYN and FAK has been well documented in various cells, especially in human neutrophils [22–25]. LYN can then interact with its downstream target, SLP-76, resulting in its tyrosine phosphorylation [26]. CD11b (integrin alpha M) is a cell surface integrin of macrophages, granulocytes, and NK cells, that becomes expressed after CD38, but before mature myeloid markers such as inducible oxidative metabolism in the process of RA-induced myeloid differentiation of HL-60 cells. It is ergo a mid-stage differentiation marker in this process. It has been reported to stimulate the phosphorylation of FAK [27]. FAK is ergo a potential mediator of CD11b signaling.

NUMB was first identified in *Drosophila*, and found to be essential for directing asymmetric cell division and development during embryogenesis. Numb mutant embryos display a dramatic loss of sensory neurons [28]. Later, it was found that the endocytosis mediated by NUMB lead to the unequal distribution of NOTCH into two daughter cells in cytokinesis, thus relating asymmetric signaling to binary cell fate choice [29]. NUMB is an evolutionarily conserved protein [30]. The mammalian NUMB contains an N-terminal phosphotyrosine-binding domain (PTB) which is required for its membrane localization, and a C-terminal Eps15 homology (EH) domain which interacts with α-adaptin and functions as an endocytic adaptor protein for clathrin-coated pit (CCP) formation and homotypic fusion of early endosomes [31–33]. We discovered that NUMB serves, too, as a scaffold protein for the RA-regulated signalosome driving differentiation of HL-60 cells. RA induced the expression of FGR which bound the signalosome resulting in enhanced phosphorylation of NUMB and NUMB-signalosome binding, in particular enhancing NUMB binding to signalosome RAF, LYN, SLP-76, and VAV, anteceding cell differentiation [15].

Here, we found that vacuolin-1, a drug that inhibits endosomal trafficking, enhanced RA-induced HL-60 cell differentiation and G1/G0 arrest. It did not enhance RA-induced expression of a marker, CD38, of early steps of differentiation, but enhanced the RA-induced expression of CD11b, as well as markers of subsequent later mature differentiation, inducible ROS and G1/0 arrest. This upregulated CD11b was associated with enhanced activation of a proposed FAK/LYN/SLP-76 signaling axis that apparently promotes later steps of RA-induced HL-60 cell differentiation. Furthermore, loss of CD11b and NUMB expression crippled late, but not early, stages of RA-induced cell differentiation. This proposed pathway suggests novel therapeutic targets for the therapy of AML. It is noteworthy that vacuolin-1 enhanced late steps of differentiation putatively through enhancing expression of CD11b but did not enhance ERK activation. But expression of CD38, an earlier induced receptor, expression of which propels RA-induced differentiation, is known to enhance ERK activation. And inhibiting ERK activation blocks differentiation. Hence ERK activation appears important for driving early stages of differentiation but not so for the late steps per se. The implication is that the process of differentiation is somewhat quantal versus continuous, which is the typical historical prejudice. These effects of vacuolin-1 are consistent with a model where RA-induced differentiation consists of progressive stages driven by the expression of sequentially induced receptors. And NUMB regulates this process. Finally the results are consistent with the conjecture that receptor endocytosis potentially regulates RA-induced differentiation.

## Materials and methods

### Cell culture

HL-60 human myeloblastic leukemia cells were a generous gift of Dr. Robert Gallagher. They are cultured in RPMI 1640 supplemented with 5% heat-inactivated fetal bovine serum (GE Healthcare, Chicago, IL) and 1% antibiotic/antimycotic (Thermo Fisher Scientific, Waltham, MA) in a 5% CO_2_ humidified atmosphere at 37  °C as previously described [34]. Retinoic acid (Sigma, St. Louis, MO) was added from a stock of 5 mM in ethanol to make a final concentration of 1 µM. Vacuolin-1 (Cayman Chemical, Ann Arbor, MI) was added from a stock of 0.25 mM in DMSO to make a final concentration of 0.25 µM. Viability assessed by 0.1% trypan blue exclusion was routinely in excess of 95% in all cultures except toxicity experiments in supplemental data that used vacuolin in excess of 2.5 µM where a sub-G1 population was also apparent.

### Construction of CRISPR-mediated stable cell lines

The CD11b and NUMB knockout cell lines were constructed as previously described with CRISPR/Cas9 [35]. The sequence for CD11b sgRNA is Forward: 5-CACCGACCTTCCAAGAGAACGCAAG-3; Reverse: 5- AAACCTTGCGTTCTCTTGGAAGGTC-3. The sequence for Numb sgRNA is Forward : 5-CACCGGATGAAGAAGGCGTTCGCAC-3; Reverse: 5-AAACGTGCGAACGCCTTCTTCATCC-3. The sg-NC control cell was the one used in our previous report [35].

### Flow cytometric phenotypic analysis

The measurements of CD38, CD11b, inducible ROS, and cell cycle analysis were performed as previously described [36]. Fluorescence was detected using a Becton Dickinson LSR II flow cytometer (San Jose, CA, USA) using 488 nm excitation. Gating to discriminate positive cells was set to exclude 95% of untreated controls.

### Western blot analysis and antibodies

The western blots were performed as previously described [35]. The antibodies used for western blots: anti- CD38 was from BD Biosciences (San Jose, CA), GAPDH, c-Raf, Phospho-c-Raf (S259), Mek, p-Mek, Erk, p-Erk, FAK, Lyn, Fgr, p47phox, Slp-76, Vav1, pY416-c-Src, horseradish peroxidase anti-mouse, and anti-rabbit antibodies, were from Cell Signaling (Danvers, MA), p-Tyr and c-Cbl antibodies were from Santa Cruz Biotechnology (Santa Cruz, CA), Phospho-c-Raf (S621) was from Thermo Fisher Scientific (Waltham, MA).

### Immunoprecipitation

Equal amount of cell lysates were precleared with Protein A/G beads for 2 h at 4  °C. The supernatant was then incubated with primary antibodies and beads overnight at 4  °C. The beads were collected and washed three times with M-PER buffer and subjected to western blot analysis. The beads were from Pure Proteome protein G magnetic beads (Millipore, Billerica, MA) and used per the manufacturer’s instructions.

### Imaging

HL-60 cells were seeded on cover glass which was precoated with Poly-L-Lysine at room temperature for 20 min followed by washing three times with PBS. After 48 h of RA or Vacuolin-1/RA treatment, 50,000 cells were collected and fixed with 4%PFA for 15 min at room temperature. The fixed cells were stained as previously described [37]. Fixed cells were blocked with PBS + 0.1% Triton-X-100 + 1% BSA for 1 h at room temperature and then incubated with primary antibody (FAK) diluted (1:1000) in blocking buffer for 2 h. After that, the cells were washed twice with PBS + 0.1% Tween-20 and incubated with secondary antibody (1:1000, Goat anti-Rabbit IgG (H + L), Alexa Fluor 488, Thermo Fisher Scientific, Waltham, MA) for 1 h, stained with 1 µg/ml DAPI at room temperature for 5 min, and then used for confocal imaging with a Zeiss LSM 880 confocal microscope.

### Statistical analysis

p-values between treatment group means were calculated using student t-test. The data represent the means of three repeats ± standard error of the mean (SEM). A p-value of < 0.05 was considered significant.

## Results

### Vacuolin-1 does not modulate RA-induced CD38 expression

**Fig. 1:**
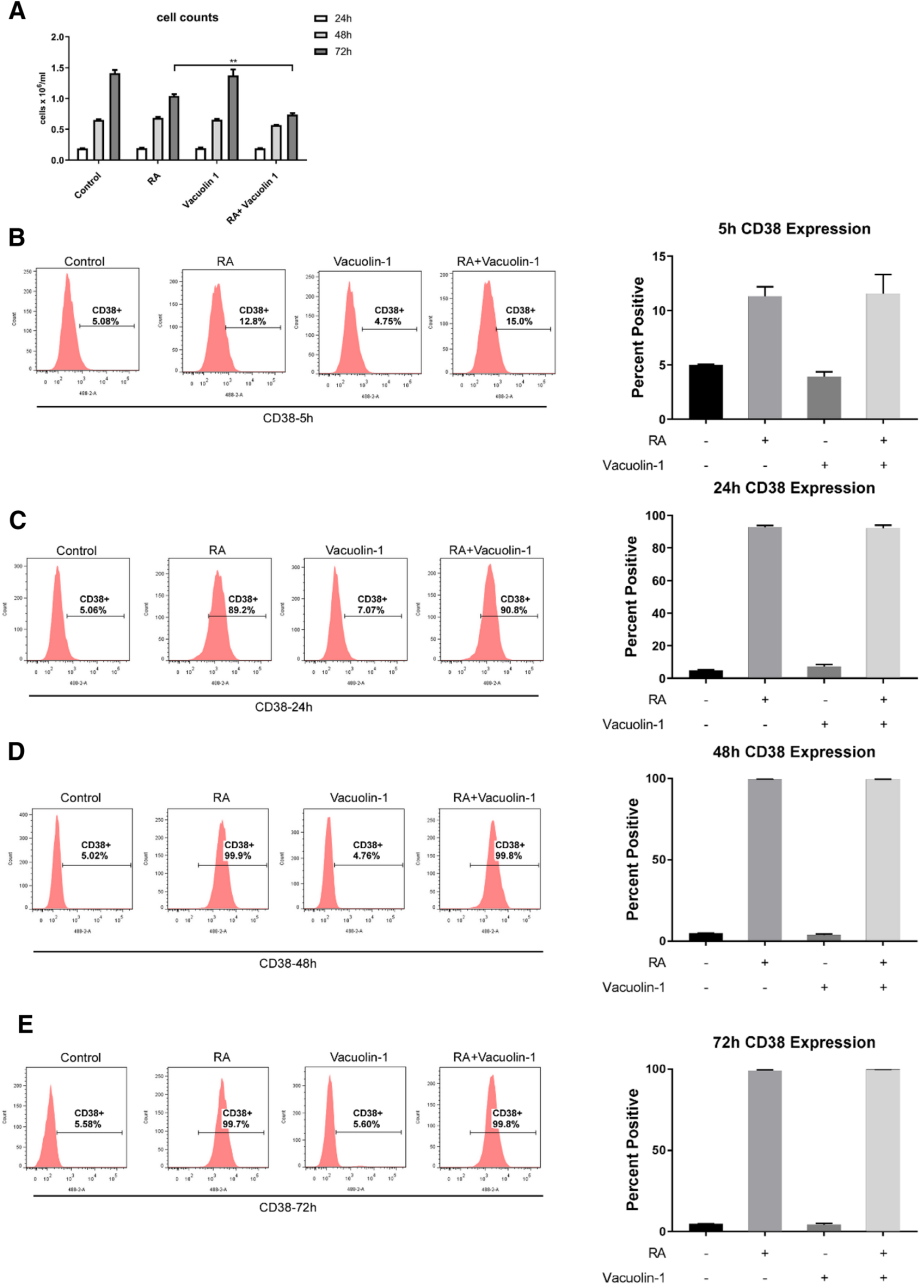
Vacuolin-1 does not modulate RA-induced CD38 expression.** A **The cell density of HL-60 cell cultures that were untreated controls or treated with 1 µM RA, 0.25 μm Vacuolin-1 or 1 µM RA plus 0.25 μm Vacuolin-1 as indicated was measured at 24 h, 48 and 72 h. **B–E** HL-60 cells were cultured for 5 h (**B**), 24 h (**C**), 48 h (**D**) and 72 h (**E**) with 1 µM RA and 0.25 µM Vacuolin-1 as indicated and membrane CD38 was analyzed using flow cytometry

To determine if vacuolin-1 could enhance RA-induced differentiation, the dose-dependent effect of vacuolin-1 on cell proliferation was determined to identify a dose that was non-toxic to use in combination with RA. We screened the optimal concentration of vacuolin-1 that was not toxic to cells using concentrations of vacuolin-1, ranging from 0.25 to 5 µM to treat HL-60 cells for 48 h. Cell population growth was measured by counting cell culture density and DNA cell cycle distribution by PI staining and flow cytometric analysis. The latter also revealed sub-G1 DNA cells betraying nuclear DNA fragmentation associated with apoptosis/death. We found that 0.25 µM did not affect cell population growth (Fig. 1A). It was well below the dose at which vacuolin-1 caused severe cell cycle disruptions, which was apparent at 5.0 µM (Additional file 1: Fig. S1). 5.0 µM caused a dramatic sub-G1 accumulation, suggesting that this concentration of vacuolin-1 induced apoptosis and was toxic. The effect was incipient at 2.5 µM. 0.25 µM vacuolin-1 was thus selected for further study with RA .

The effect of vacuolin-1 on RA-induced CD38 was determined. CD38 expression is the earliest telltale that the process of RA-induced differentiation has begun [38]. Its ectopic expression generates MAPK pathway signaling and accelerates induced differentiation. To minimize any overt toxic effects by vacuolin-1, 0.25 µM vacuolin-1 was used to ascertain whether it can promote RA-induced HL-60 cell differentiation. Cells were either untreated, treated with RA, vacuolin-1 or vacuolin-1 plus RA, and expression of CD38 was measured by flow cytometry. RA induced CD38 expression and adding 0.25 µM vacuolin-1 to the 1 µM RA did not further increase the percentage of CD38 positive cells measured at 5 h, 24 h, 48 and 72 h **(**Fig. 1B–E). These results indicate that vacuolin-1 did not affect early steps of RA-induced differentiation marked by CD38 expression, which is consistent with the known prominent RARE locate in the first intron that drives CD38 expression [39, 40].

### Vacuolin-1 enhanced RA-induced expression of CD11b

**Fig. 2:**
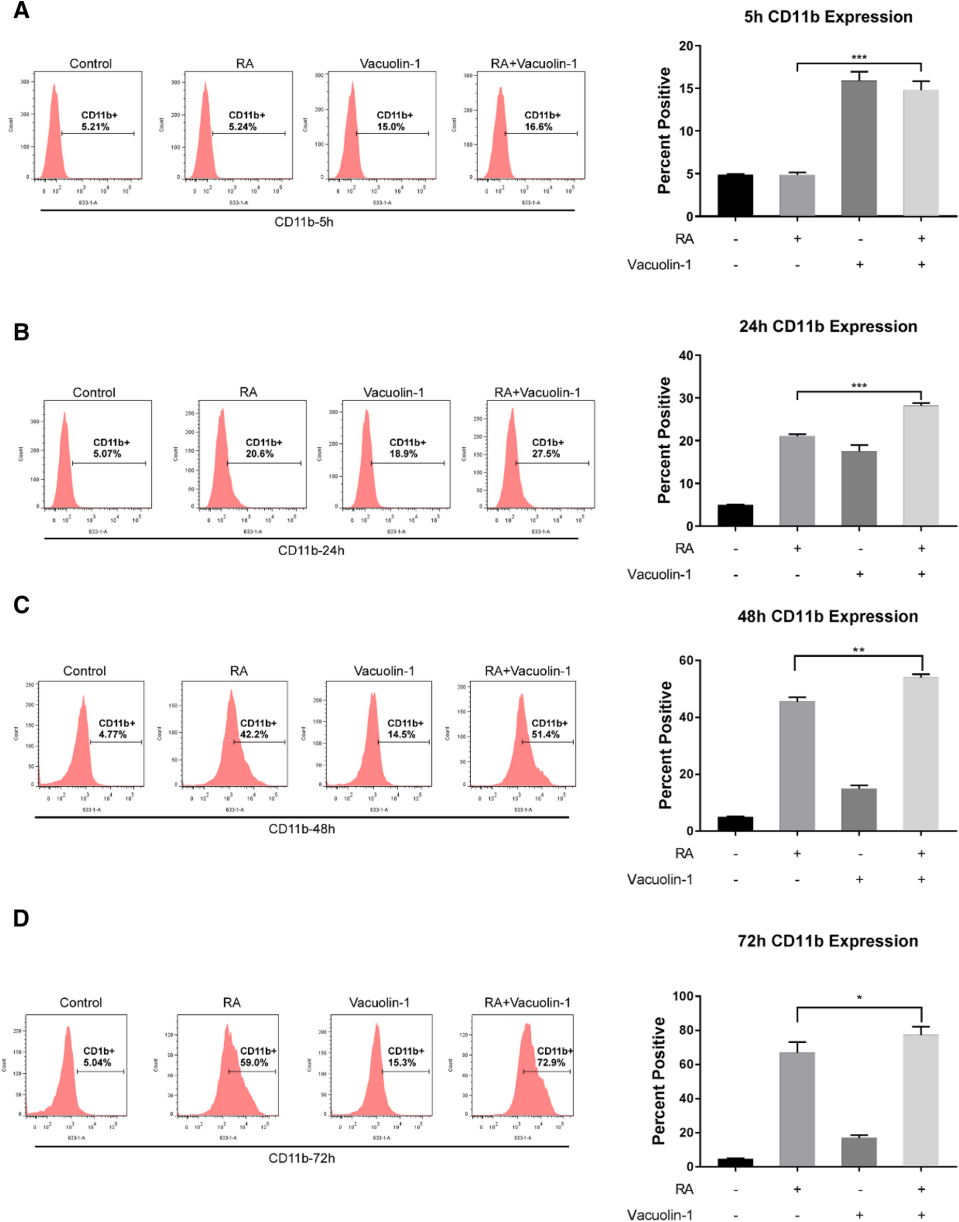
Vacuolin-1 enhances RA-induced expression of CD11b. **A**–**D** HL-60 cells were cultured for 5 h (**A**), 24 h (**B**), 48 h (**C**) and 72 h (**D**) with 1 µM RA and 0.25 µM Vacuolin-1 as indicated and membrane CD11b was analyzed using flow cytometry. Control is untreated

The effect of vacuolin-1 on RA-induced CD11b was determined. The expression of CD11b is a mid-stage differentiation marker induced by RA after CD38 and before manifestation of inducible oxidative metabolism or cell cycle arrest characteristic of mature cells [41]. CD11b is encoded by *ITGAM* and dimerizes with CD18 to form the functional integrin heterodimer, CD11b/CD18, also known as alpha-M beta-2 (αMβ2) [42]. In macrophages, CD11b is required for the activation of SRC and SYK kinases [43]. Cells were untreated, treated with RA, vacuolin-1 or RA plus vacuolin-1, and CD11b expression was measured by flow cytometry after 5 h, 24 h, 48 and 72 h of culture. Co-treatment with vacuolin-1 and RA significantly increased the percentage of CD11b positive cells compared to RA alone that was detectable at 24 h, 48 and 72 h (Fig. 2). Vacuolin-1 thus enhanced expression of this mid-stage marker in the process of RA-induced differentiation. Anecdotally, we noted, too, that in another human myeloid leukemia cell line, NB4, the only other well known established one, vacuolin-1 also enhanced RA-induced CD11b expression (Additional file 1: Fig. S2).

### Vacuolin-1 enhanced RA-induced markers of late steps of the differentiation into mature cells: inducible oxidative metabolism and G1/0 cell cycle arrest

**Fig. 3:**
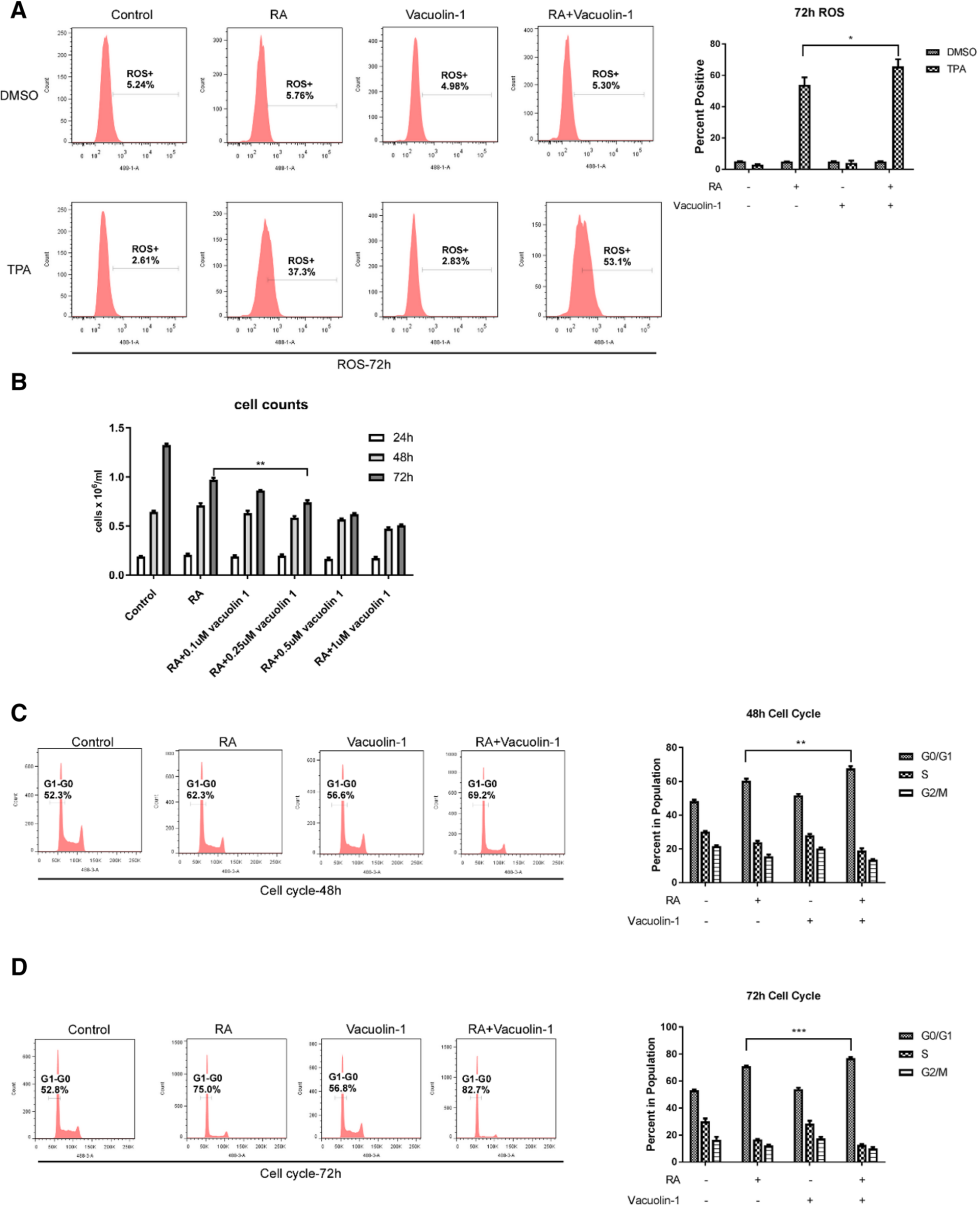
Vacuolin-1 enhances RA-induced markers of late steps of the differentiation into mature cells: inducible oxidative metabolism and G1/0 cell cycle arrest.** A** HL-60 cells were cultured for 72 h with 1 µM RA and 0.25 µM Vacuolin-1 as indicated, where Control is untreated, and the inducible ROS was analyzed using flow cytometry. DMSO is the carrier control for TPA stimulation. **B** The cell density of HL-60 cell cultures that were untreated controls or treated with 1 µM RA or RA plus different (0.1, 0.25, 0.5 and 1.0 µM) concentrations of Vacuolin-1 as indicated was measured at 24 h, 48 and 72 h. **C**, **D** HL-60 cells were cultured for 48 h (**C**) and 72 h (**D**) with 1 µM RA and 0.25 µM Vacuolin-1 as indicated and the cell cycle phase distributions were analyzed using flow cytometry. Control is untreated

The effect of vacuolin-1 on RA-induced functional differentiation, which characterizes late steps of induced differentiation, was determined. Functional differentiation was detected by inducible oxidative metabolism betrayed by TPA-induced production of reactive oxygen species (ROS). ROS is a functional differentiation marker of mature differentiated cells. Cells were either untreated, treated with RA, vacuolin-1 or vacuolin-1 plus RA. Vacuolin-1 in combination with RA enhanced the percentage of cells positive for TPA-induced reactive oxygen species (ROS) production compared with RA alone **(**Fig. 3A**)**. Vacuolin-1 thus enhanced RA-induced expression of the functional marker of mature cells.

The effect of vacuolin-1 on RA-induced growth inhibition via G1/0 cell cycle arrest was determined. Growth inhibition characterizes mature differentiated cells. Cells were either untreated, treated with RA, vacuolin-1 or vacuolin-1 plus RA. Vacuolin-1 enhanced RA-induced inhibition of population growth (Fig. 3B ). Enhanced growth inhibition due to vacuolin-1 was apparent at 72 h in mature cell populations. The effect of vacuolin-1 on RA-induced G1/0 cell cycle arrest corroborated this. G1/0 cell cycle arrest betrayed by the accumulation of G1/0 DNA cells is characteristic of mature differentiated cells. Cells were either untreated, treated with RA, vacuolin-1 or vacuolin-1 plus RA; and their cell cycle distribution was measured by propidium iodide staining and flow cytometry after 48 and 72 h of culture. Vacuolin-1 enhanced the RA-induced enrichment of G1/0 cells, consistent with retarding cell population growth (Fig. 3C, D). Vacuolin-1 thus enhanced RA-induced markers characteristic of mid to late steps of differentiation into mature cells, namely CD11b and subsequently inducible ROS and growth arrest associated with G1/0 accumulation. The results suggest that expression of CD11b drives later events in the process of RA-induced differentiation and make its signaling attributes in this context of interest.

### Vacuolin-1 modulates a CD11b/FAK/LYN/SLP-76 signaling axis to promote RA-induced HL-60 cell differentiation

Adding vacuolin-1 to RA treatment enhanced RA-induced upregulation of CD11b, with ensuing enhancement of subsequent steps of differentiation. To determine if enhanced CD11b expression attributable to Vacuolin-1 had signaling consequences potentially relevant to driving differentiation, activation of signaling molecules downstream of CD11b that are known drivers for differentiation was measured. CD11b is a member of the integrin family of receptors known to act through FAK and activate MAPK related signaling pathway components. Lyn is a member of the family of Src-Like Family Kinases (SFK) which are known to associate with FAK [44]. Lyn is a component of the signalosome known to be activated by RA. It associates with other signalosome components including the adaptor, SLP-76. Expression of SFKs and adaptors such as SLP-76 or CBL, are known to propel differentiation [17, 18, 38, 45, 46]. This motivates the speculation that activation of a CD11b/FAK/LYN/SLP-76 signaling axis is involved. Accordingly we tested if vacuolin-1 contributed to enhancing RA-induced activation of these signaling molecules. Cells were untreated or treated with RA, vacuolin-1, or vacuolin-1 plus RA for 48 h. Immunoprecipitates using the signaling molecule as bait were resolved by Western blotting and probed for p-tyr.

**Fig. 4:**
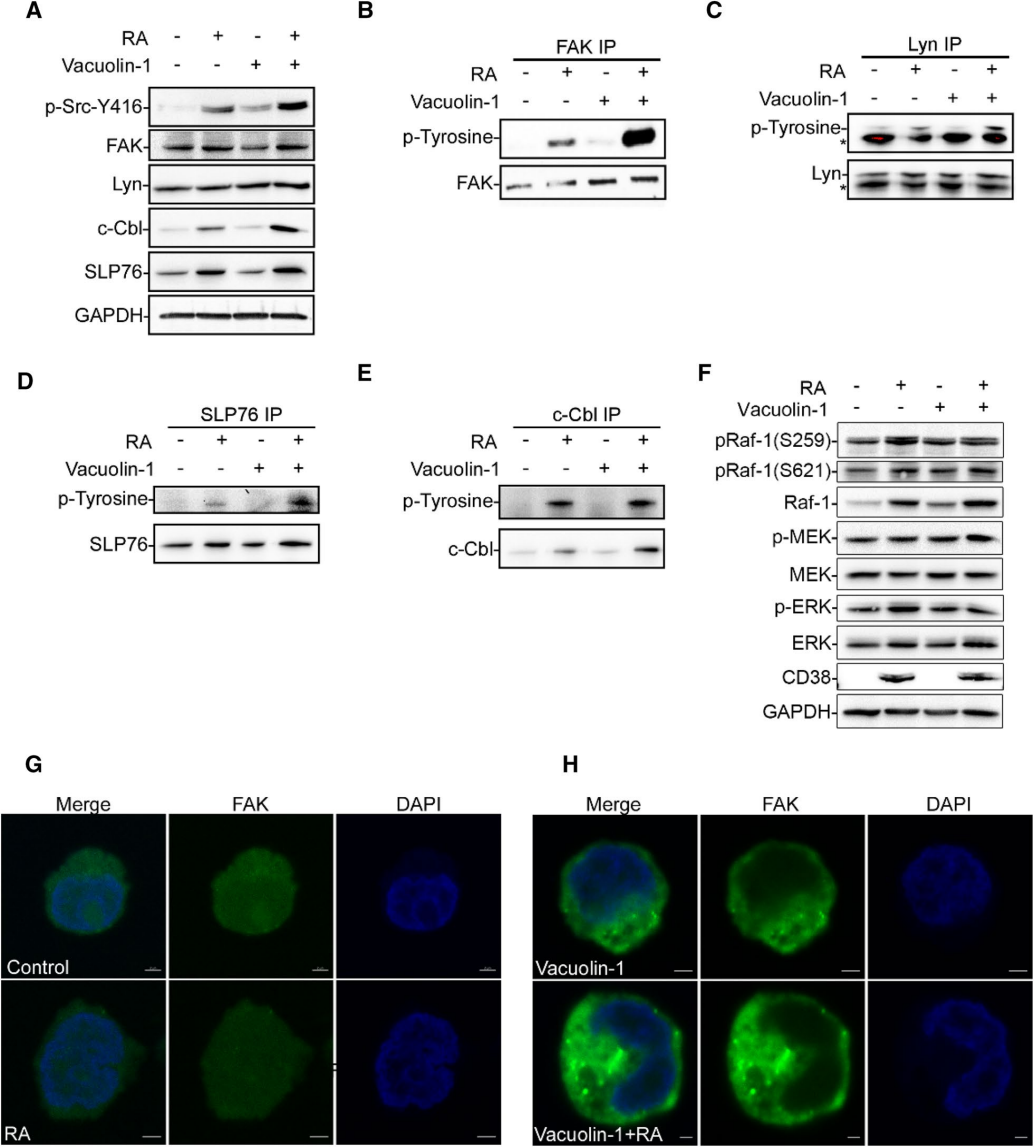
Vacuolin-1 modulates a CD11b/FAK/LYN/SLP-76 signaling axis to promote RA-induced HL-60 cell differentiation. **A** HL-60 cells were cultured for 48 h with 1 µM RA and 0.25 µM Vacuolin-1 as indicated (Control is untreated), the cell lysates were collected, and the indicated proteins were analyzed by western blot for molecules indicated, Y416 phosphorylated SFKs, FAK, LYN, c-CBL, SLP-76. GAPDH is the loading control. **B**–**E** HL-60 cells were cultured for 48 h with 1 µM RA and 0.25 µM Vacuolin-1 as indicated, the cell lysates were collected, and FAK immunoprecipitates (IP) (**B**), Lyn IP (**C**), Slp-76 IP (**D**) and c-cbl IP (**E**) were analyzed by Western blots probed with anti-p-tyr antibody. **F** HL-60 cells were cultured for 48 h with 1 µM RA and 0.25 µM Vacuolin-1 as indicated, the cell lysates were collected, and the indicated proteins were analyzed by western blot, probing for the indicated molecules. **G**, **H** HL-60 cells were untreated controls or 1 µM RA-treated **G** or 0.25 µM Vacuolin-1 or 0.25 µM Vacuolin-1 plus 1 µM RA-treated **H** for 48 h as indicated, the cells were collected and stained for FAK for confocal microscopy. DAPI was used to stain nuclear DNA and delineate the nucleus

RA caused the tyrosine phosphorylation of FAK, and this was enhanced by addition of vacuolin-1 to the RA treatment. Likewise LYN tyrosine phosphorylation was also enhanced. The phosphorylation level of Y416 SFKs, which is the telltale phosphorylation event for SFK activation, was measured, and vacuolin-1 enhanced RA-induced LYN activation. The primary phosphorylated SFK in HL-60 cells is Lyn [47]. SLP-76, an adaptor molecule in the signalosome that is associated with LYN, also became tyrosine phosphorylated after RA treatment and addition of vacuolin-1 enhanced this **(**Fig. 4A–D**)**. LYN and SLP-76 are constituents of the NUMB scaffolded signalosome that RA activates to drive differentiation. NUMB is a membrane protein that is known to be tyrosine phosphorylated in response to RA [15]. By contrast RA-induced tyrosine phosphorylation of CBL, another putative adaptor in the signalosome which is also associated with integrin signaling [48], was apparently not affected by addition of vacuolin-1. Nor was activation of the RAF/MEK/ERK axis imbedded in the signalosome **(**Fig. 4E F**)**, indicating that the vacuolin-1 effects appear somewhat specific and were not catholic for all signalosome adaptors or components. Western blots **(**Fig. 4A**)** showed that the expression levels of FAK, LYN, and SLP-76 remained similar when comparing the vacuolin-1-RA co-treatment group with the RA-only group, while c-CBL expression was enhanced after vacuolin-1 cotreatment with RA. The data are ergo consistent with the proposition that CD11b drives a novel CD11b/FAK/LYN/SLP-76 signaling axis subject to endocytic regulation that propels RA-induced HL-60 cell myeloid differentiation.

These results motivate interest in whether vacuolin-1 might affect the intracellular relocalization of FAK. Confocal microscopy was used observe the intracellular distribution of FAK. Fixed cells were immunofluorescently stained for FAK and DAPI stained for DNA. In control and RA-treated cells FAK is relatively unformly diffusely distributed in the cell **(**Fig. 4G**)**. Vacuolin-1-treated cells by contrast showed an apparent redistribution of FAK toward the periphery of the cell consistent with an association with the CD11 cell surface receptor **(**Fig. 4H**)**.

### Deletion of CD11b or Numb attenuates RA-induced HL-60 cell differentiation

**Fig. 5:**
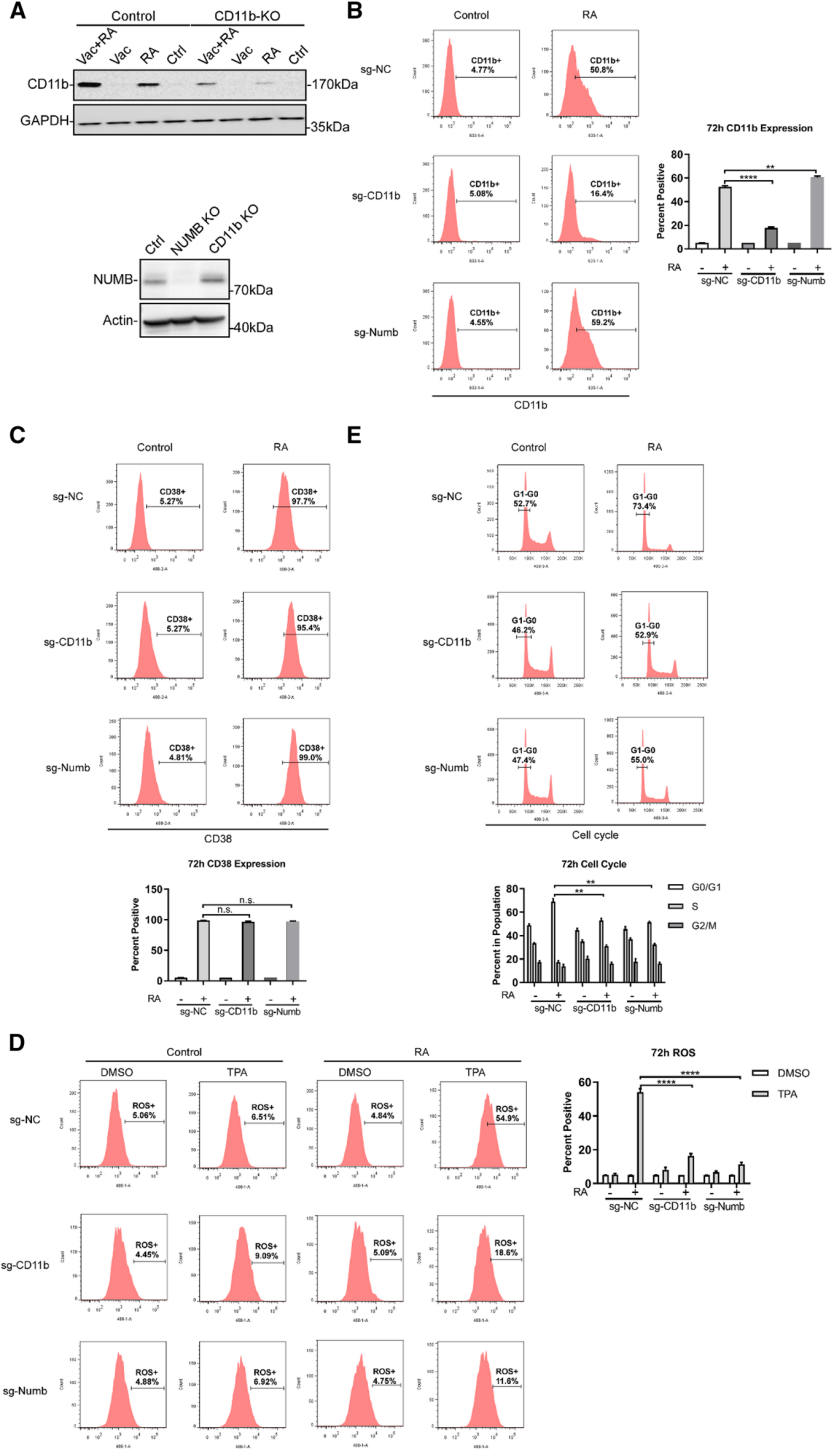
Deletion of CD11b or Numb attenuates RA-induced HL-60 cell differentiation. **A** The sg-NC (non-specific guide control), sg-CD11b (CD11b targeted CRISPR KO) and sg-Numb (Numb targeted CRISPR KO) stably transfected HL-60 cells were collected for WB analysis of CD11b and Numb. **B** The sg-NC, sg-CD11b and sg-Numb HL-60 cells were cultured for 72 h with or without (Control) 1 µM RA as indicated and membrane CD11b was analyzed using flow cytometry. **C–E** The sg-NC, sg-CD11b and sg-Numb HL-60 cells were cultured for 72 h with or without 1 µM RA as indicated, and membrane CD38 (**C**), inducible ROS induced by TPA **D** and G1/G0 cell cycle arrest **E** were analyzed as indicated by flow cytometry. DMSO is the carrier control (the negative) for TPA (the positive) used to induce oxidative metabolism (Reactive Oxygen Species, ROS)

The present results on the effects of vacuolin-1 on RA-induced differentiation suggest that the expression of CD11b induced by RA drives subsequent late steps in the process of differentiation. To test this proposition CD11b expression was crippled by CRISPR KO intervention. Stable transfectants were created using CD11b directed and non-specific control guides using pooled transfectants to obviate clonal bias. Untreated or RA-treated control and CD11b CRISPR/cas9 targeted transfectants were analyzed for CD11b expression after 72 h of culture. Western blotting showed that RA induced CD11b expression in the non-specific guide control typical of wt parental cells and that addition of vacuolin-1 enhanced this, but RA-induced expression in the CD11b targeted transfectants was grossly crippled and still greatly crippled with addition of vacuolin-1 (Fig. 5A). Flow cytometric analysis of immunofluorescently labelled CD11b expression corroborated that the expression in RA-treated CD11b targeted cells was greatly crippled, but not in the non-specific control (Fig. 5B). Both assays detected a very small amount of CD11b expression in RA-treated CD11b targeted cells that is unexplained, but may reflect a small subpopulation of cells that survived despite prolonged selection; however, both assays also demonstrate that the population of CD11b targeted cells was largely crippled in their ability to express CD11b. RA-induced CD38 expression was unaffected by loss of CD11b. Both the control and CD11b targeted transfectants expressed CD38 with 100% of the cells positive at 72 h (Fig. 5C). Expression was measured by flow cytometry of immunofluorescenly stained cells. In contrast, RA-treated CD11b targeted cells failed to express the functional differentiation marker, inducible oxidative metabolism, detected by ROS production, whereas the control cells did express this at a level typical of wt parental cells (Fig. 5D). The inducible oxidative metabolism was measured by flow cytometry to detect ROS production of TPA-stimulated cells. Like inducible ROS production, another marker of late steps in RA-induced differentiation marking mature cells, G1/0 cell cycle arrest was also crippled by loss of CD11b. While control cells showed RA-induced enrichment of G1/0 cells characterizing G1/0 cell cycle arrest, the CD11b targeted cells failed to do so (Fig. 5E ). We note anecdotally, too, that absent CD11b, vacuolin fails to enhanced RA-induced phosphoY416 marked LYN activation, consistent with the above posted CD11b driven LYN activation (Additional file 1: Fig. S3). .Loss of CD11b thus resulted in cells unable to undergo late steps of RA-induced differentiation, namely inducible oxidative metabolism and G1/0 cell cycle arrest, but were competent for early steps, namely CD38 expression.

The present results suggest that NUMB has a potentially important role in RA-induced differentiation, as a regulator of the signalosome that generates signals to enable RA-driven transcription and of endocytosis that regulates the signaling. To further study its involvement in RA-induced HL-60 cell differentiation, stable transfectants with depleted Numb were created with CRISPR/Cas 9. Western blotting showed that endogenous NUMB expression was ablated (Fig. 5A). To determine the effect of losing NUMB on RA-induced differentiation, CD38, CD11b, ROS and G1/0 cell cycle arrest were measured. Loss of Numb did not affect RA-induced expression of CD38 **(**Fig. 5C**)**. Nor did it cripple induced expression of CD11b, which was actually somewhat enhanced as might be expected if CD11b endocytosis were impaired (Fig. 5B). However, RA-induced inducible ROS production (Fig. 5D) and G1/G0 cell cycle arrest (Fig. 5E) were grossly crippled by loss of NUMB. Hence, these data implicate an essential role for Numb in regulating RA-induced HL-60 cell differentiation. However, that role appears complicated because of potentially multiple functions that NUMB performs.

## Discussion

RA, an active metabolite of vitamin A1, mediates the functions of vitamin A1 required for growth and development. Cell proliferation and differentiation are informed by extracellular signals. One such class of signals is from integrin cell surface receptors. Signaling by activated cell surface receptors is governed by receptor endocytosis. Endocytosis can terminate or revise their signaling. Vacuolin-1 is a small molecule that leads to the accumulation of early endosome through disrupting early endosome to late endosome transition, motivating interest in its effect on RA-induced differentiation. RA-induced HL-60 cell differentiation involves the progressive manifestation of CD38, CD11b, inducible ROS production and G1/0 arrest in mature cells. Here we found that RA-induced phenotypic conversion with progressive CD11b, inducible ROS production and G1/0 cell cycle arrest is enhanced by Vacuolin-1 treatment **(**Figs. 1, 2 and 3**)**. Enhanced CD11b was associated with enhanced phosphorylation/activation of FAK, LYN and SLP-76, suggesting a possible CD11b/FAK/LYN/SLP-76 axis supporting differentiation, where Vacuolin-1 enhances this signaling by interrupting endocytic mediated CD11b receptor degradation. Consistent with this, losing expression of CD11b, the start of the axis, inhibits RA-induced G1/0 cell cycle arrest and ROS, two late markers of the differentiation process characteristic of fully mature cells, while induced CD38 expression is unaffected. **(**Figs. 4 and 5**)**. In this paradigm, RA induces expression of CD11b which contributes propulsion to differentiation through a putative CD11b/FAK/LYN/SLP-76 axis that drives late stages of differentiation and is subject to regulation by endosomes. This signaling may use the LYN and SLP-76 of the signalosome that is potentially localized at the plasma membrane by Numb and thereby contributes to sustaining a prolonged signalosome activation/signaling needed to elicit differentiation.

Historically integrin family receptors recruit FAK and SRC kinases to effect various cellular processes, such as migration, lamellipodium formation [49], angiogenesis [50], and tumor growth [51], rooted in MAPK pathway activation via phosphorylation. Here we report that it cooperates with RA to control leukemic cell differentiation and proliferation through tyrosine phosphorylation activation of a FAK/LYN/SLP-76 signaling axis **(**Fig. 4A–D**)**. Because LYN and SLP-76 are components of the signalosome that generates signals to enable RARE-regulated transcription, one can speculate that CD11b exerts this control through regulation of the function of the signalosome, which is acting as a potential signal integrator as well as generator in a possible feedback loop. Indeed such regulatory feedback has been found for RA, although its nature was not fully defined at the time [11].

LYN is a Src family kinase member that is a signalosome component up-regulated by RA [47, 52]. LYN interacts with other RA-upregulated molecules, SLP-76 and CD38, which belong to the signalosome, to enable RA-induced HL-60 cell differentiation [18, 53]. Moreover, by utilizing SFK inhibitor PP2, it has been found that the kinase activity of LYN promotes the RA-induced assembly of this CD38 regulated signalosome, but not the phosphorylation of ERK [18]. Here we found that Vacuolin-1 treatment increased the phosphorylation level of both LYN and SLP-76 upon RA treatment, consistent with the notion that CD11b uses this signaling axis to regulate the signalosome and hence the signals that propel differentiation.

Vacuolin-1 is one of the 6-morpholino-1,3,5-triazine derivatives, which is a class of endosome trafficking inhibitors. It targets capping protein Zβ (CapZβ) to inhibit endosome trafficking, and also significantly suppresses breast tumor metastasis [54]. Here we found that Vacuolin-1 promoted RA-induced HL-60 cell differentiation in a manner consistent with the integrin/FAK/LYN/SLP-76 signaling axis. Interestingly, while enhancing CD11b expression, Vacuolin-1 treatment did not significantly affect the protein level of FAK, LYN or SLP-76, but enhanced the RA-induced phosphorylation level of these targets **(**Fig. 4A–D**)**. Moreover, Vacuolin-1 alone also enhanced the expression of CD11b **(**Figs. 2 and 5A**)**. This is consistent with an earlier report that CD11b is expressed in untreated HL-60 cells at a very low level [55], so vacuolin-1 might enhance its abundance by inhibiting endosome trafficking.

To further verify the role of endocytic CD11b in RA-induced HL-60 cell differentiation, we depleted CD11b with CRISPR/Cas9. Consistent with the hypothesized role of CD11b as the trigger for the activation of the FAK/LYN/SLP-76 signaling axis to drive late steps of differentiation, loss of CD11b cripples RA-induced ROS production and cell cycle arrest. Although there is a report that cell cycle arrest is not needed for RA-induced CD11b, as our kinetics also indicate, it appears here that CD11b loss cripples RA-induced cell cycle arrest [56]. It should be noted from the flow cytometry analysis, that there is still a small positive population of CD11b left in the sg-CD11b cells when treated with RA for 72 h **(**Fig. 5A**)**. We do not know the cause of this. The sg-CD11b cells are a pool instead of a single clone, and some untargeted cells may have somehow survived several rounds of selection; or possibly the CRISPR associated transcriptional pause block may leak, too [57]. The use of single clone might result in a complete CD11b knockout, but the use of a pool obviates the possibility of clonal bias. Regardless, it does not compromise the conclusion that loss of CD11b cripples late steps of differentiation.

Finally, we tested the effect of loss of NUMB, using a CRISPR KO, on RA-induced differentiation. HL-60 cells undergoing proliferation express NUMB, and NUMB is a scaffold for a signaling complex, signalosome, that when activated by FGR provides signals to enable RA-induced transcriptional activation needed for cell differentiation [15]. At the same time, however, NUMB functions as an adaptor in the formation and homotypic fusion of early endosomes. Using RNAi to knockout Numb leads to clustered but unfused early endosomes [33]. Consistent with NUMB involvement in receptor endocytosis, we found that flow cytometry revealed higher membrane CD11b in the knockout than that in control sg-NC cells **(**Fig. 5B**)**; i.e., consistent with impaired endocytosis-mediated degradation of CD11b following the loss of Numb. Numb KO, however, also impaired late steps of RA-induced cell differentiation, namely inducible ROS production and G1/0 arrest, albeit not expression of CD38, a marker of an earlier differentiation step. This is consistent with a posited role for NUMB in effecting RA-induced differentiation. However, knocking down NUMB and vacuolin-1 both target endocytosis, but with different effects on differentiation—vacuolin-1 enhances, whereas loss of NUMB cripples, differentiation. This is consistent with the existence of different mechanisms, namely via the signalosome or early endosome formation, whereby NUMB can regulate differentiation. The total loss of NUMB thus probably impairs signalosome function and thereby impairs differentiation. Thus, importantly, the knockout ergo shows that NUMB regulates differentiation, but that NUMB function in regulating RA-induced differentiation reflects more than one regulatory process, where positive and negative regulation are balanced.

## Supplementary Information

**Additional file 1: Figure S1.** The cell cycle profiles for the HL-60 cells treated with indicated concentration of Vacuolin-1 for 48 h. Hypotonic propidium iodide stained samples were analyzed by flow cytometry. Control is untreated cells. **Figure S2.** NB4 cells were cultured for 72 h with 1 µM RA and 0.25 µM Vacuolin-1 as indicated and the CD38 (**A**), CD11b (**B**) and cell cycle phase distributions **C** were analyzed using flow cytometry. Control is untreated. **Figure S3.** Wild type and CD11b KO cells were culture for 72 h with 1 µM RA and 0.25 µM vacuolin-1 as indicated and analyzed by Western blots probed for phospho Y416, the activating phosphorylation of Src-Family-Kinases. RA induces pY416, which we posited is phospho-Y416 LYN [47], and adding vacuolin-1 to RA enhances this, as shown in Results, but without CD11b, the vacuolin-1 no longer enhances; i.e., RA induces pY416 LYN that is enhanced by addition of vacuolin, but the enhancement is CD11b dependent as it fails to occur without CD11b. This is consistent with the thesis advanced that vacuolin-1, an inhibitor of receptor endocytosis, acts at least in part to enhance later events in differentiation by enhancing CD11b expression to drive differentiation-promoting signaling—specifically LYN activation
