# Correction: Vacuolin‑1 enhances RA‑induced differentiation of human myeloblastic leukemia cells: evidence for involvement of a CD11b/FAK/LYN/SLP‑76 axis subject to endosomal regulation that drives late differentiation steps

**DOI:** 10.1186/s13578-023-00965-0

**Published:** 2023-02-03

**Authors:** Kaiyuan Zhu, Noor Kazim, Jianbo Yue, Andrew Yen

**Affiliations:** 1 Department of Biomedical Sciences, Cornell University, Ithaca, NY USA; 2 Department of Biomedical Sciences, City University of Hong Kong, Hong Kong, China; 3 Division of Natural and Applied Sciences, Synear Molecular Biology Lab, Duke Kunshan University, Kunshan, China; 4 City University of Hong Kong Shenzhen Research Institute, ShenZhen, China

**Correction:** **Cell & Bioscience (2022) 12:179 **10.1186/s13578-022-00911-6

In this article, the affiliations details for the author Kaiyuan Zhu was incorrectly given as 3, 4 but it should be 1, 2, 4 and for the author Jianbo Yue was incorrectly given as 1, 2, 4 but it should be 3, 4. And also, the email address for Prof. Jianbo Yue is jianbo.yue@duke.edu.

The original article [1] has been corrected.
