# Supplementary material for: Vacuolin-1 enhances RA-induced differentiation of human myeloblastic leukemia cells: evidence for involvement of a CD11b/FAK/LYN/SLP-76 axis subject to endosomal regulation that drives late differentiation steps
Source: Cell Biosci. 2022 Nov 3;12:179. doi: 10.1186/s13578-022-00911-6 (PMC9635152; doi:10.1186/s13578-022-00911-6)
Supplement: Supplementary file 1 — Additional file 1: Figure S1. The cell cycle profiles for the HL-60 cells treated with indicated concentration of Vacuolin-1 for 48 h. Hypotonic propidium iodide stained samples were analyzed by flow cytometry. Control is untreated cells. Figure S2. NB4 cells were cultured for 72 h with 1 µM RA and 0.25 µM Vacuolin-1 as indicated and the CD38 (A), CD11b (B) and cell cycle phase distributions C were analyzed using flow cytometry. Control is untreated. Figure S3. Wild type and CD11b KO cells were culture for 72 h with 1 µM RA and 0.25 µM vacuolin-1 as indicated and analyzed by Western blots probed for phospho Y416, the activating phosphorylation of Src-Family-Kinases. RA induces pY416, which we posited is phospho-Y416 LYN [47], and adding vacuolin-1 to RA enhances this, as shown in Results, but without CD11b, the vacuolin-1 no longer enhances; i.e., RA induces pY416 LYN that is enhanced by addition of vacuolin, but the enhancement is CD11b dependent as it fails to occur without CD11b. This is consistent with the thesis advanced that vacuolin-1, an inhibitor of receptor endocytosis, acts at least in part to enhance later events in differentiation by enhancing CD11b expression to drive differentiation-promoting signaling—specifically LYN activation [file 13578_2022_911_MOESM1_ESM.docx]

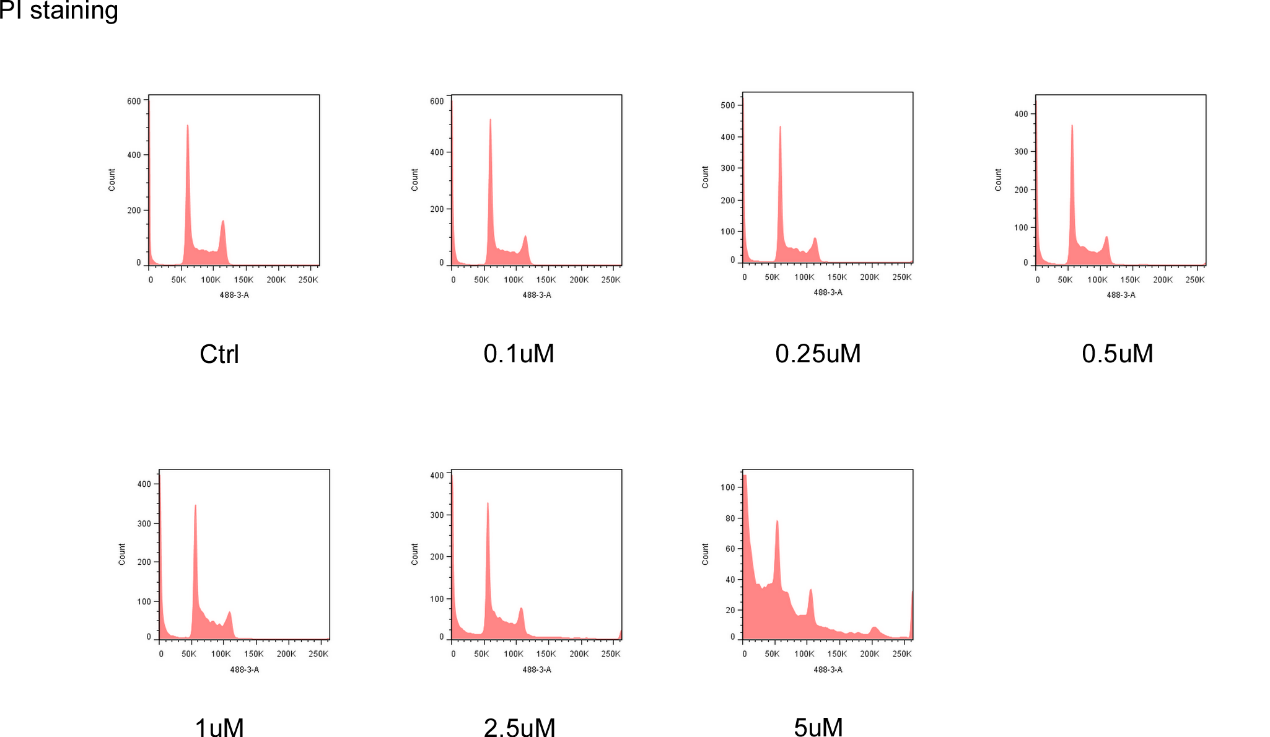


**Figure S1.** The cell cycle profiles for the HL-60 cells treated with indicated concentration of Vacuolin-1 for 48h. Hypotonic propidium iodide stained samples were analyzed by flow cytometry. Control is untreated cells.


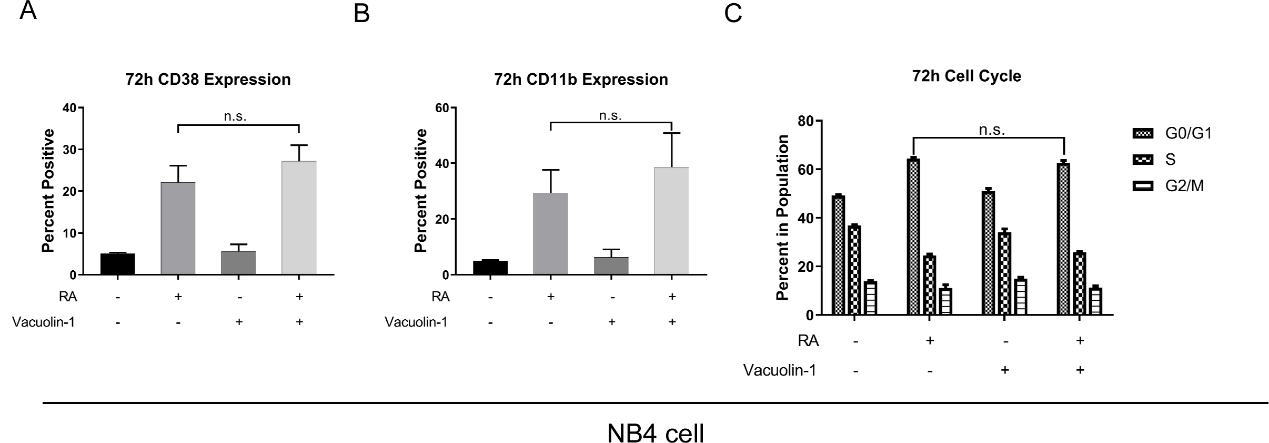
**Figure S2.** NB4 cells were cultured for 72h with 1μM RA and 0.25 μM Vacuolin-1 as indicated and the CD38 **(A)**, CD11b **(B)** and cell cycle phase distributions **(C)** were analyzed using flow cytometry. Control is untreated.


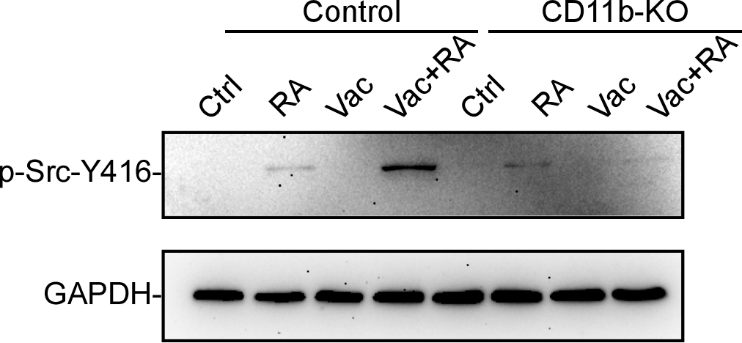


**Figure S3.** Wild type and CD11b KO cells were culture for 72h with 1 μM RA and 0.25 μM vacuolin-1 as indicated and analyzed by Western blots probed for phospho Y416, the activating phosphorylation of Src-Family-Kinases. RA induces pY416, which we posited is phospho-Y416 LYN[47], and adding vacuolin-1 to RA enhances this, as shown in Results, but without CD11b, the vacuolin-1 no longer enhances; ie., RA induces pY416 LYN that is enhanced by addition of vacuolin, but the enhancement is CD11b dependent as it fails to occur without CD11b. This is consistent with the thesis advanced that vacuolin-1, an inhibitor of receptor endocytosis, acts at least in part to enhance later events in differentiation by enhancing CD11b expression to drive differentiation-promoting signaling – specifically LYN activation.
